# Multiple Perforations along the Entire Colon as a Complication of Intestinal Behcet's Disease: A Rare Case

**DOI:** 10.4103/1319-3767.39625

**Published:** 2008-04

**Authors:** Umit Sekmen, Tolga Muftuoglu, Julide Sagiroglu, Ozgun Gungor

**Affiliations:** Department of General Surgery, Haydarpasa Numune Education and Training Hospital, Istanbul, Turkey

**Keywords:** Colonic perforation, intestinal Behcet's disease, ulcer

## Abstract

Colonic complications of Behcet's disease due to intestinal involvement are rarely reported in the literature. Ulcers are the most frequently seen intestinal complications that cause bleeding and perforation predominantly in the ileocecal region. In this article, we report a patient with Behcet's disease who presented with multiple perforations along the entire colon. Postoperative histopathological examination revealed multiple ulcers containing lymphocytic infiltrations in the small peripheral and submucosal venules. Intimal thickening and fresh intraluminal fibrin thrombosis were also seen in these venules.

Behcet's disease (BD) is a rare, chronic, and lifelong disorder presenting with inflammation of blood vessels throughout the body. This disorder may also cause various types of skin lesions, arthritis, bowel inflammation, and meningitis. Additionally, intestinal, hepatic, splenic, and pancreatic involvement rarely occur during the course of the disease.[1,2] Intestinal ulcerative changes are seen in <1% of patients with BD and ileocecal region is the most frequent site of gastrointestinal (GI) involvement. Rarely, other sites of the colon may also be involved. Few studies have reported colonic perforations due to BD in the literature. In this article, we report the case of a patient with BD presenting with multiple perforations along the entire colon.

## CASE REPORT

A 31-year-old male complaining of severe abdominal pain and nausea was admitted to the emergency room of our hospital. Physical examination revealed a rigid, distended, clearly tender abdomen on palpation, and rebound tenderness was positive at all quadrants. Laboratory investigation revealed white blood cell count 19000/mm^3^, hemoglobin 16 mg/dl, glucose 102 mg/dl, urea 65 mg/dl, and creatinine 1.55 mg/dl. An upright abdominal X-ray demonstrated free air under the diaphragms bilaterally and intraabdominal hyperintense free fluid was clearly seen in abdominal ultrasonography.

At the age of 22, he was diagnosed with BD because of his oral and genital ulcers as well as left ankle arthritis, and a medical treatment regimen with oral ointments and colchicine was started. After 3 weeks, he was symptom free and stopped taking his drugs. Within the first 10 years of initial diagnosis of BD, he had a drug-free period except for the irregular occasions that he mentioned taking colchicine. Interestingly, in this period, he had episodes of oral and genital ulcers, which disappeared spontaneously without any treatment. He had never been followed up by any healthcare facility on a regular basis. He was positive for HLA B5 and pathergy test was negative. With two major and two minor criteria, he was diagnosed with incomplete BD according to the diagnostic criteria of the International Study Group for BD.[3] Both his brother and sister were diagnosed with BD as well.

After the initial evaluation in the emergency room, he was diagnosed for acute abdomen and we immediately took him to the operating room. Laparotomy revealed massive intraabdominal purulent fluid, multiple perforations, and extensive inflammation in all the segments of the colon. Subtotal colectomy with drainage was our choice of surgical treatment at that point.

**Figure 1:**
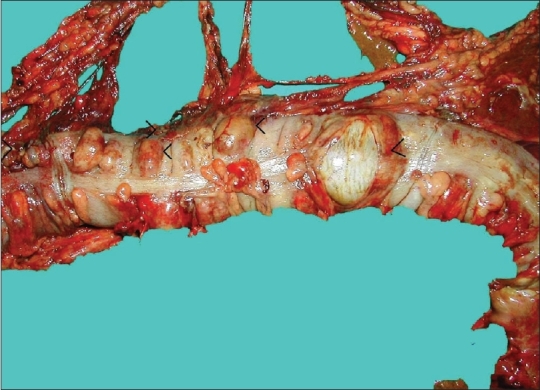
Multiple penetrations (<) and perforation (>) in transverse colon

**Figure 2:**
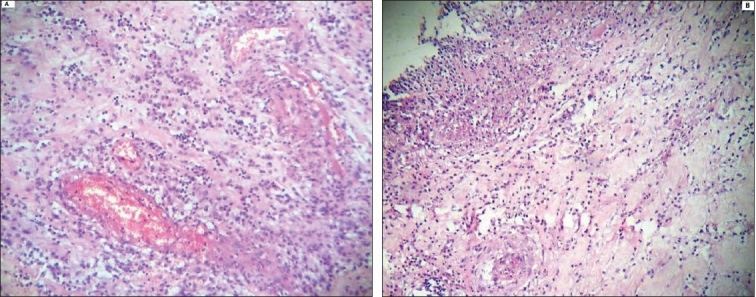
A: Small vessels demonstrating intimal thickening and lymphocytic vasculitis; B: Submucosa underlying an ulcer containing nonspecific chronic inflammation

Pathological analysis of the resected specimens demonstrated multiple round and oval mucosal ulcers with sharp margins and measuring <3 cm in diameter. Many of these ulcers were penetrated and perforated, and surrounded with extensive edema [Figure 1]. Peripheral and submucosal small venules underlying these ulcers demonstrated intimal thickening and contained lymphocytic infiltration as well as intraluminal fresh fibrin thrombosis. The other features observed were submucosal edema, fibrosis, focal lymphocytic aggregation, and polymorphonuclear leukocytic infiltration through the serosa [Figure 2].

## DISCUSSION

Patients with BD are considered for surgical intervention as soon as their GI ulcers become the source of hemorrhage, pain, or inflammatory mass. Pain is the most frequently seen complication of BD ulcers (92% of the cases).[4] Ulcers of the colon in BD are mostly localized in the ileocecal region. Involvement of the other parts of the lower GI system is very rare.[3,5–11] Incidence of GI involvement of BD varies according to the geographical location.

According to the available data, ulcer perforation and penetration in patients with this disease have been both found to occur at similar rates (50%).[1,12] Kasahara ***et al***. reported that 73% of patients with enteric BD had multiple ulcers, of which 76% had localized ulcers (frequently around terminal ileum).[1,2,12] Perforations of the colon are very rare except for the ileocecal region. Two cases of sigmoid perforation[6,7] have been reported in the literature. Since BD and its colonic involvement are rare, misdiagnosis is common.

Such is the rarity of colonic ulcers in patients with BD that their development in such patients should prompt a widening of the differential diagnosis to include Crohn's disease, ulcerative colitis, steroidal or nonsteroidal anti-inflammatory drug-induced ulcers, and amoebiasis.[4] Intestinal lesions of Crohn's disease tend to appear as longitudinal ulcers with cobblestone appearance and, noncaseating epitheloid granulomas, resulting in a narrowed and more rigid colonic segment. In ulcerative colitis, lesions usually start from rectum, tend to be diffuse with extensive mucosal inflammation with intervening normal mucosa. Lesions due to NSAIDs may mimic ulcers in BD; they may manifest as pseudomembranous colitis, collagenous colitis, as well as a single bleeding ulcer located in the proximal colon. Lesions due to amoebiasis may also closely mimic BD; however, they may be distinguished by demonstrating amoebae in stool specimens.

Intestinal ulcers of BD are usually round or oval with a “punched out” appearance mainly located in the ileocecal region and are deeper than those in ulcerative colitis. They may rarely display granulomatous features and longitudinal appearance (only three cases have been reported). With vasculitis, especially venulitis, the intestine in BD usually has a normal intervening mucosa and relatively normal wall thickness.[2,4,5,8–10,12] In our case, we resected nearly 90 cm of colon, from the ileocecal ring to the rectosigmoid region. Multiple ulcers were observed in all the segments of the colon, several of which were penetrated and perforated. We could not find a similar case in the literature. It will be crucial to keep in mind that multiple colonic perforations may be caused by intestinal ulcers of BD.

In summary, intestinal BD, when diagnosed, can be treated with two alternative methods—conservative (medicines and follow up) or surgery. Management modalities of intestinal ulcers of BD are still controversial. However, a generally accepted approach is conservative treatment, unless any of the disease-related complications necessitates surgical intervention.[12,13] In contrast, a few experienced authors prefer surgery in the treatment of intestinal BD due to high complication risk and poor response to conservative methods. More cases need to be overseen to gain more experience in the correct treatment of this disease.
